# The Test of Conduct and Difficulty of Definition

**Published:** 1915-07-03

**Authors:** Bryan Donkin


Conference (*continued*).

### SIR BRYAN DONKIN'S ADDRESS.

### The Test of Conduct and Difficulty of Definition.

The President, Sir Bryan Donkin, M.D., taking the chair after the Lord Mayor's departure, gave the following address.

### Aim of the Conference.

This Conference has been convened in recognition of the need felt by most persons who are responsible for the working of the Mental Deficiency Act for a better understanding and consensus (1) on the usage of the descriptive terms established by that Act; and (2) on the most efficient means (*a*) of distinguishing the so-called "mentally deficient" from the so-called normal, (*b*) of duly grouping children generally believed to be mentally deficient, for the purpose of such education and treatment as may be desirable. The intention of this Conference is thus exclusively practical, and is not concerned with the scientific question of the causation of mental deficiency, or with the fitness, or otherwise, of such descriptive terms for the various groups of mentally deficient persons as have now been established by the Mental Deficiency Act. I would therefore ask all speakers in this discussion to confine themselves as strictly as possible to the subjects just mentioned, and merely remind anyone who may be tempted to approach too nearly the quicksands of debate on "Heredity" that, whatever divergences of opinion may be latent in the minds of those here, we are, most of us, if not all, convinced that what we all agree to call "mental deficiency" tends in a marked degree (to use a clear and popular phrase) to "run in families." We can thus approach without hesitation the really practical difficulties that we have to consider.

I will now note shortly some of the difficulties encountered by the different usage of the descriptive terms in the Act, and, indeed, of the very title of the Act itself. Not only adverse critics who have no personal experience of the subject-matter, but also others who, from their studies and experience, might be expected to be more accurate, erroneously use the term "mental" as interchangeable with "intellectual," and from this terminological confusion proceed to argue that "mental deficiency" is not an adequate basis for differentiating from the normal large numbers of those who will require care under the Act. I would, therefore, point out that in the descriptions of all the various classes of persons to be dealt with under the Act, the test of conduct in human association is made prominent, and pre-eminently so by the separate place, co-ordinate with the classes of idiots, imbeciles, and feeble-minded, accorded, not, certainly, for logical, but for strictly practical purposes, to the important clinical group of so-called "moral" imbeciles. It cannot be too often insisted on that moral deficiency or ineducability is included under mental deficiency or ineducability, and that the observation of "defective conduct," as has been so explicitly and repeatedly taught by Dr. Mercier, is the essential basis of the inference of insanity or of mental deficiency in any individual case.

Another misuse of terms is met with in the loose employment of the word "feeble-minded," both to cover all grades of mental deficiency and also to denote one grade only. It has been largely the custom, apparently for the purpose of euphemism, for American writers to use this term in the general sense; but an attempt has been recently made by some of them to minimise the resulting confusion by the introduction of the Greek term "moron" to designate the highest grade of the mentally defective. It would appear, however, that the test of moronism rests on the degree of intelligence only, so that the American "moron" is not identical with the "feeble-minded" as indicated in the English Mental Deficiency Act.

It is among the class of the feeble-minded, according to this Act, that are found by far the most persons who, from their defective conduct, are the most harmful to the community, and most urgently require due recognition for the purpose of care and control. Logically, indeed, the "moral imbecile" is a sub-class of the feeble-minded, but he could not always be caught in the net cast for "morons." Further difficulties arising from the equivocal or erroneous use of terms may probably be furnished in the course of the subsequent discussion.

### Who are the Feeble-Minded?

Coming now to the next subject of the Conference—viz., the best means of differentiating the "mental defective" from the general mass of those who, whether correctly or not, are usually and intelligibly called "normal," it is clear that the real question for us is the differentiation of the class known as "feeble-minded" from the normal; for "imbeciles" and "idiots" as described in the Act offer no difficulty to the diagnostician or to the administrators of the law.

Much unnecessary difficulty has been raised, both before and after the passing of the Mental Deficiency Act, by erroneous and pedantic assumptions, made in diverse and mainly non-medical quarters, that "mental deficiency" is a clear-cut entity, capable of strictly limited definition, and of being handled as such by biologists, statisticians, and jurists. And these assumptions are made whether the term "mental deficiency" is taken to mean defective intellect alone, or, in the proper sense, defect of mind generally as evidenced by clinical study and observation of conduct. For illustration of this I would refer to two works of widely differing character which have recently appeared: the one is by H. H. Goddard, Ph.D., of New Jersey, entitled, "Feeble-Mindedness: Its Causes and Consequences," in which feeble-mindedness is regarded as due to the absence of normal intelligence, and normal intelligence is assumed to be a "unit character" transmitted in true Mendelian fashion; the other is by Professor

**Conference** (*continued*).

Karl Pearson, entitled "The Graduated Character of Mental Defect, and the Need for Standardising Judgments as to the Grade of Social Inefficiency which shall involve Segregation."

Of these authors, the first adopts the Binet measuring scale of intelligence as the standard for differentiation of the "morons," or the highest grade of mental defectives, from the normal; the second appears to expect some method of standardising judgments on grades of social inefficiency, but gives no indication of the way to arrive at it.

It is clear, I think, to most who have any personal knowledge and experience of this subject, that for legal and practical purposes we can no more look for strict definitions or a hard-and-fast line between the "feeble-minded" and the "normal" than for a similarly rigid division between the normal and the insane; and that in both cases the diagnosis is certainly not a matter of the intelligence only. It is a matter of "defect of mind" in all as evidenced chiefly by careful and often prolonged observation of conduct, and by study of the history of each case, leading to an inference of the incapacity of the subjects to adjust themselves effectively to their social surroundings. In some cases there will even be inevitable differences of opinion; but, even in these, prolonged observation will usually overcome the difficulty of coming to a just decision.

On the last point indicated at the outset of my remarks—viz., the best means of differentiating from the normal such children as are suspected of being either "backward" or mentally defective, and of properly grading them when so differentiated, I do not propose to enlarge now, as the papers to be read, especially those on the Binet method, will supply full material for debate. This discussion will doubtless be concerned mainly with the question of how far any attempt to make general the use of a prescribed standardisation of intelligence is likely to assist practically in the actual diagnosis of mental deficiency, or in making clear in a Law Court the grounds of opinion given by a medical witness when questions of mental deficiency are raised.

